# A Study of Dielectric Properties of Proteinuria between 0.2 GHz and 50 GHz

**DOI:** 10.1371/journal.pone.0130011

**Published:** 2015-06-12

**Authors:** Peck Shen Mun, Hua Nong Ting, Teng Aik Ong, Chew Ming Wong, Kwan Hong Ng, Yip Boon Chong

**Affiliations:** 1 Department of Biomedical Engineering, Faculty of Engineering, University of Malaya, Kuala Lumpur, Malaysia; 2 Department of Surgery, Faculty of Medicine, University of Malaya, Kuala Lumpur, Malaysia; 3 Department of Medicine, Faculty of Medicine, University of Malaya, Kuala Lumpur, Malaysia; 4 Department of Biomedical Imaging, Faculty of Medicine, University of Malaya, Kuala Lumpur, Malaysia; 5 Damansara Specialist Hospital, Petaling Jaya, Selangor, Malaysia; Hospital Universitario de La Princesa, SPAIN

## Abstract

This paper investigates the dielectric properties of urine in normal subjects and subjects with chronic kidney disease (CKD) at microwave frequency of between 0.2 GHz and 50 GHz. The measurements were conducted using an open-ended coaxial probe at room temperature (25°C), at 30°C and at human body temperature (37°C). There were statistically significant differences in the dielectric properties of the CKD subjects compared to those of the normal subjects. Statistically significant differences in dielectric properties were observed across the temperatures for normal subjects and CKD subjects. Pearson correlation test showed the significant correlation between proteinuria and dielectric properties. The experimental data closely matched the single-pole Debye model. The relaxation dispersion and relaxation time increased with the proteinuria level, while decreasing with the temperature. As for static conductivity, it increased with proteinuria level and temperature.

## Introduction

The measurement of dielectric properties is generating interest for clinical utility. Dielectric properties of tissues have been widely determined to provide informative data to the literature related to the application of measurements for biological dielectric properties. Surowiec et al. [1] measured the dielectric properties of animal tissues following the time of tissue death in radio frequency. They concluded that the concentration of cell-derived ions in tissues exhibits changes in dielectric properties. Recently, studies have been conducted in microwave frequencies due to stronger observable interaction with polarization of ions. Gabriel et al. [2] and Gabriel et al. [3] reviewed the studies of differences in dielectric properties among biological tissues and further comparative measurement changes with tissue type, biological fluid, temperature and frequency, respectively. They reported that frequency dependence of human and animal tissues corresponded to the temperature of tissue extraction at frequencies up to 20 GHz. Dielectric behavior was affected by water content and blood infiltration within the tissues [2, 3]. Previous studies related to biological fluids reported biomaterial dependency of dielectric changes. Variations in cell type, hematocrit, ionic salt and glucose level in blood resulted in changes to dielectric properties, respectively [4–8]. Peyman and Gabriel [9] found that the differences in dielectric properties between porcine and ovine bile at 37°C were due to the differences in composition of bile between the two species.

The chemical compounds and biomaterials drastically affect the electrical properties of a solution. Boresch et al. [10] and Matyushov [11] reported frequency dependence in simulated dielectric properties between the interaction of protein and water. The presence of protein causes the change in dielectric properties as it alters the mobility and linear conduction of a solution [6, 12]. Oncley [13] compared the dielectric properties of different serum protein solutions with pure water. The dielectric properties increased with the concentration of carboxyhemoglobin solution at radio frequencies. Ferry and Oncley [14] discovered that serum and urinary albumin protein molecules had relatively faster electrophoresis mobility than other proteins, which resulted in the increment of the dielectric constant. They pointed out that the dielectric constant of the serum protein increases with the concentration at the radio frequency. However, Nandi and Bagchi [15] found out that the dielectric constant increased with whale myoglobin solution at low frequency and decreased at high frequency. In another study, Grant et al. [16] reported that there was a relatively good change in dielectric dispersions of different protein concentrations with temperature. The dielectric constant of bovine serum albumin decreased with temperature for frequencies less than 1 GHz. Besides that, the dielectric constant decreased with the concentration of bovine serum albumin at 700 MHz.

Proteinuria is a condition characterized by the presence of protein in urine. It is one of the early signs of kidney disease. Persistent proteinuria followed by progressive decline of renal function (increments in serum creatinine level) are presentations of chronic kidney disease (CKD) and this eventually leads to end stage renal disease [17]. Although tests for proteinuria may not be applicable to all CKD patients, the presence of proteinuria has been identified in patients with increased risk of kidney disease progression [18, 19]. Diabetes mellitus and hypertension are among the most common causes of chronic kidney disease, apart from other clinical conditions such as congestive heart failure, pyelonephritis, polycystic kidney disease, glomerulonephritis and autoimmune disorder (systemic lupus erythematosus) [20]. Monitoring of urinary protein is required as standard care in the diagnosis and prognostication of patients with chronic kidney disease.

Previous studies had investigated the dielectric properties of protein solutions from animals, such as amino acids [10, 21], horse hemoglobin [13, 14], bovine serum albumin [16], whale myoglobin [15], and lysozyme from chicken egg [22]. So far, no studies have been conducted to look into the dielectric properties of protein in urine, extracted from subjects with CKD. No data has been reported for changes in the dielectric properties of proteinuria with temperature. Furthermore, no measurements have been carried out to determine the effects of different proteinuria levels on dielectric properties, as well as the correlation between proteinuria levels and dielectric properties. In this paper, we investigated the dielectric properties of urine in normal subjects and in subjects with CKD at room temperature (25°C), at 30°C and at body temperature (37°C), respectively, between microwave frequency of 0.2 GHz and 50 GHz. We found significant changes in urine dielectric properties with proteinuria and temperature, respectively. Significant correlation between proteinuria levels and dielectric properties was reported. The experimental data closely matched the single-pole Debye model.

## Materials and Methods

### Urine collection and Storage

A total of 83 subjects were recruited in this study. Out of these, 43 subjects with chronic kidney disease (disease duration > 3 years) were under medical care at the University of Malaya Medical Centre (UMMC). The other 40 subjects were recruited as the control group, which involved normal healthy subjects. The characteristics of the subjects are shown in Table 1. This study was approved by the Institutional Ethics Review Committee, UMMC, Malaysia. All subjects provided written informed consent to participate in this study.

**Table 1:** Characteristics of the subjects.

Subject	Normal	CKD
**Total (N)**	40	43
**Gender (Male/ Female)**	17/23	14/29
**Age (years)**	30 ± 3	66 ± 21

The characteristics of recruited subjects are shown.

Urine samples consisting of 60 ml random spot mid-stream urine were collected from each subject. The urine samples were collected using sterile urine containers. Fresh urine samples were stored in the refrigerator at a temperature of 4°C within 4 h before the measurement of dielectric properties was conducted. No preservatives were added to the urine upon collection.

### Urine Composition and Measurement

The urine composition of each subject was measured using routine methods at the Division of Laboratory Medicine, UMMC to determine the urine clinical chemical variables in terms of protein, glucose, hemoglobin, creatinine, urea and salt (Cl^−^, Na^+^ and K^+^) content. Subjects whose urine showed the presence of glycosuria and hematuria were excluded from this study.

### Measurement Setup

The measurement system for dielectric properties consists of: (1) Agilent E8364C personal network analyzer (PNA; 10 MHz-50 GHz) operated with Agilent 85070 software through Agilent 82357A GPIB interface (Agilent Technologies, Santa Clara, CA); and (2) 50 GHz flexible cable with open-ended coaxial slim probe designed by Agilent Technologies for liquids and semi-solid materials. The PNA was calibrated with references for air, short circuit and deionized water before measurements took place. Electronic-calibration (E-Cal) was used as the standard for refresh calibration. Random and systemic errors were taken into account to reduce uncertainties as reported by Gabriel and Peyman [23].

**Fig 1:**
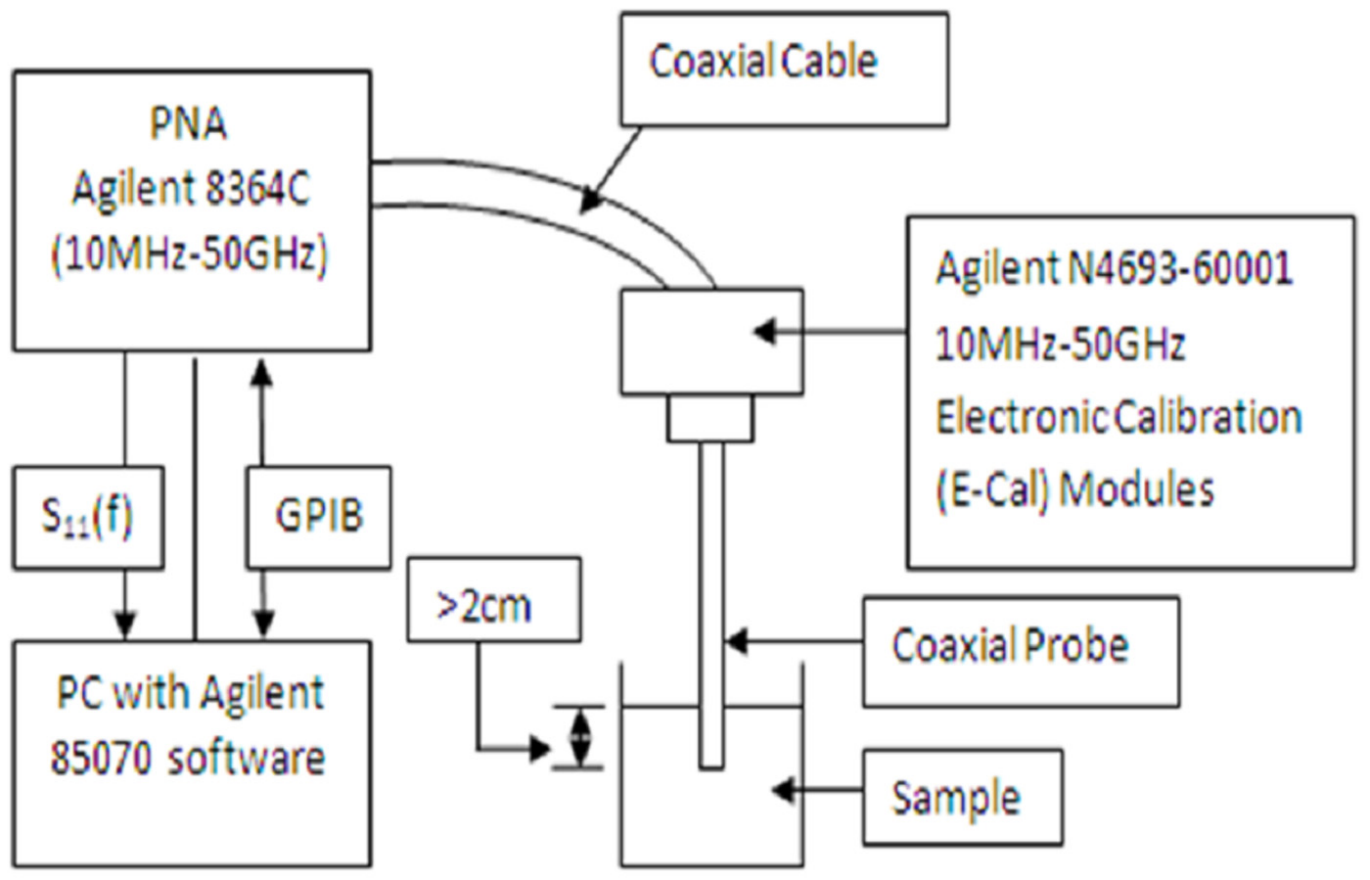
Schematic representation of the measurement setup. Agilent E8364C personal network analyzer (PNA; 10 MHz-50 GHz) operated with Agilent 85070 software through Agilent 82357A GPIB interface was used to measure dielectric properties of urine samples. Reflection coefficient (S_11_) measured by PNA was converted to dielectric properties of the urine sample by the program software. Electronic-calibration (E-Cal) module provided the standard refresh calibration for measurement.

Before measurements were conducted, the urine samples were heated to room temperature (25°C) using a water bath (Memmert WNB7, Duesseldorf, Germany) with a precision of ± 0.1°C and the samples were gently stirred. Movement of the test table and probe was avoided by adjusting the sample to the probe. The measurements were taken when the probe was immersed >2 cm with perfect contact and with no air bubbles under the probe tip (Fig 1). The probe was sterilized using alcohol wipes and cleaned with distilled water before each measurement. Experiments were repeated by heating the urine sample to 30°C and 37°C, respectively. For each experiment, three measurements were recorded with each urine sample for frequencies ranging from 0.2 GHz to 50 GHz.

### Data Analysis

Dielectric properties, in terms of dielectric constant (ε’) and dielectric loss factor (ε”), were obtained from the measurements at the microwave frequency range from 0.2 to 50 GHz. A total of 250 frequency points were measured with an interval of 200 MHz. The one-way analysis of variance (ANOVA) test was conducted to determine the effect of temperature on the dielectric properties at different microwave frequencies. The Independent Samples T test was used to determine the statistically significant differences in dielectric properties and urine composition between normal and CKD subjects. Pearson correlation test was conducted to determine correlation between proteinuria levels and dielectric properties. The level selected for statistical significance was set at probability value <0.05. All the statistical analysis tests were carried out using SPSS Statistic 21.0 program (SPSS, Inc., Chicago, IL).

### Curve Fitting

In polar liquids, each type of polar molecule exhibits a particular characteristic response to an imposed electric field. Dielectric relaxation is the delay of molecular polarization with the change of the electric field in electromagnetic frequencies. Theoretically, Debye model describes the wideband frequency dependence of the dielectric relaxation response [12, 24]. Single Debye relaxation shows a good representation of water over temperature ranging from 0–100°C for frequencies up to 50 GHz [25]. Single-pole Debye equation, as below, was applied to fit the experimental data over the frequency range of between 0.2 GHz and 50 GHz using Matlab fitting function:

$$\varepsilon (\omega )=\varepsilon_{\infty }+\frac{\Delta \varepsilon }{1+j\omega \tau }-j\frac{\sigma_{s}}{\varepsilon_{0}\omega }$$ (1)

where ε(ω) is the complex relative permittivity (dielectric properties) and ω is the angular frequency. Infinite frequency permittivity (ε_∞_), magnitude of dispersion (Δε), relaxation time (τ), static conductivity (σ_s_) are the parameters of the variables to fit the experimental data. Limits such as ε_∞_ ≥ 1, Δε ≥ 0, σ_s_ ≥ 0 and τ ≥ 0 were set on the fitting parameters so that they would remain within physical ranges.

The fitting analysis was conducted using a genetic algorithm (GA) to compute function score of complex curve-fitting program with iterations. GA performs direct search optimized parameters with best fitness from a population. The level of population size was selected at 1500 with cross over fraction, 0.5 was set. The program calculates the root mean square percentage error (RMSPE) between the differences of experimental value and the value obtained from the model for fitting. The data were fitted independently for each subject group at the respective temperature.

## Results and Discussion

### Urine composition

**Fig 2:**
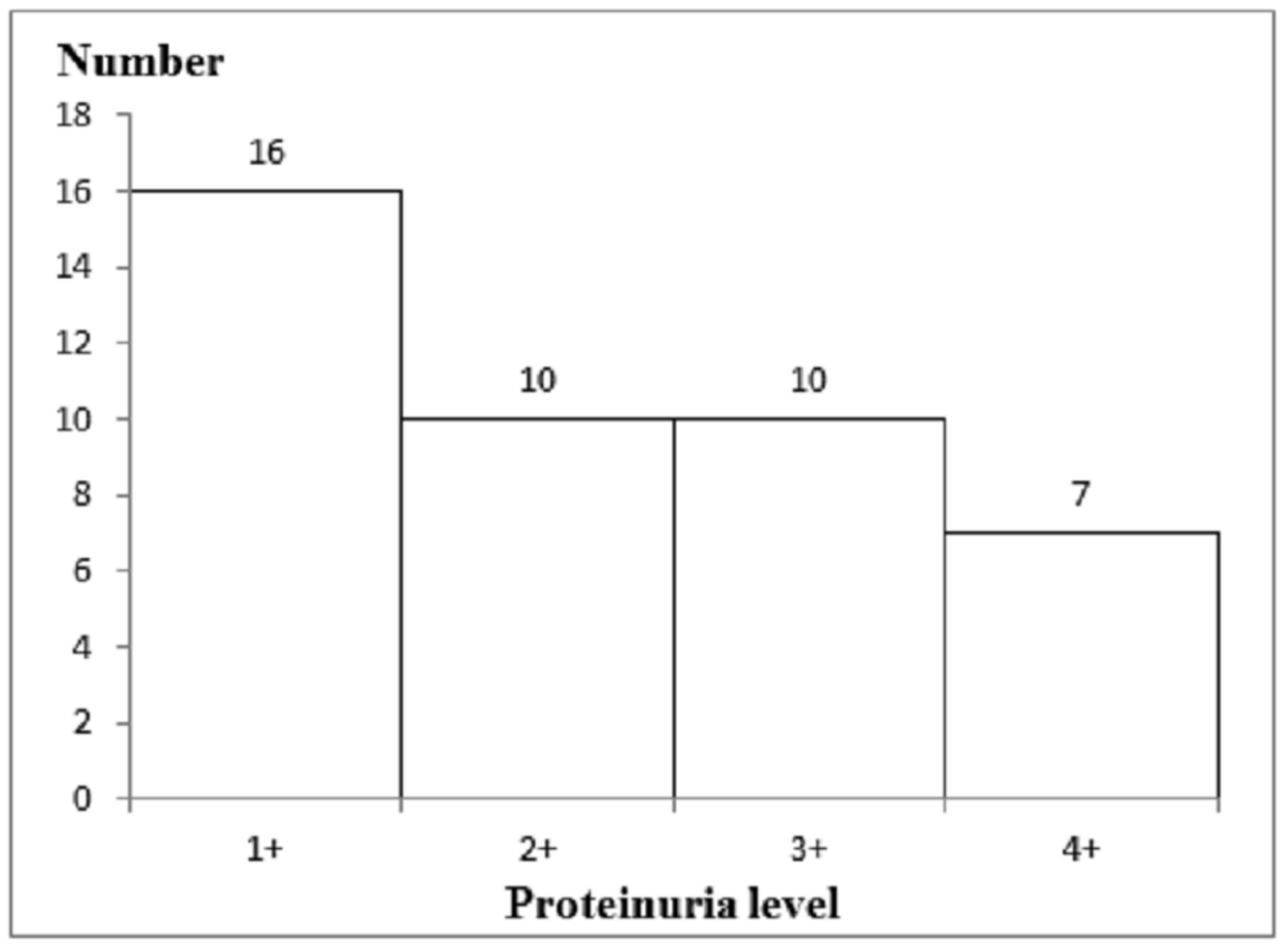
Number distribution of CKD subjects in urinalysis proteinuria levels. We further determined the number of CKD subjects according to their urinalysis proteinuria levels: 1+ (0.25g/L), 2+ (0.75g/L), 3+ (1.5g/L) and 4+ (5g/L), respectively.

Lower levels of creatinine, urea and salt ions were found in CKD compared with normal subjects (Table 2). Proteinuria was found in CKD subjects. This could be explained by the fact that CKD patients suffer from kidney malfunction, which affects the body’s efficiency in removing waste and causes leakage of protein into the urine. The distribution number of CKD subjects based on their urinalysis proteinuria levels: 1+ (0.25g/L), 2+ (0.75g/L), 3+ (1.5g/L) or 4+ (5g/L) are presented in Fig 2.

**Table 2:** Chemical variables of normal and CKD subjects.

Chemical Variables	Normal	CKD
**Creatinine (μmol/L)**	8846 ± 2677	6032 ± 1951
**Urea (mmol/L)**	183 ± 25	118 ± 46
**Cl** ^**−**^ **(mmol/l)**	103 ± 66	85 ± 33
**Na** ^**+**^ **(mmol/l)**	90 ± 59	83 ± 31
**K** ^**+**^ **(mmol/l)**	32 ± 26	25 ± 13

The urine chemical variables of each recruited normal and CKD subject were determined using routine methods at the Division of Laboratory Medicine, UMMC. The data presented were the mean urine chemical variables of normal (n = 40) and CKD (n = 43) subjects ± standard deviation (S.D.) quantity of measured chemical variables in urine.

### Overview

**Fig 3:**
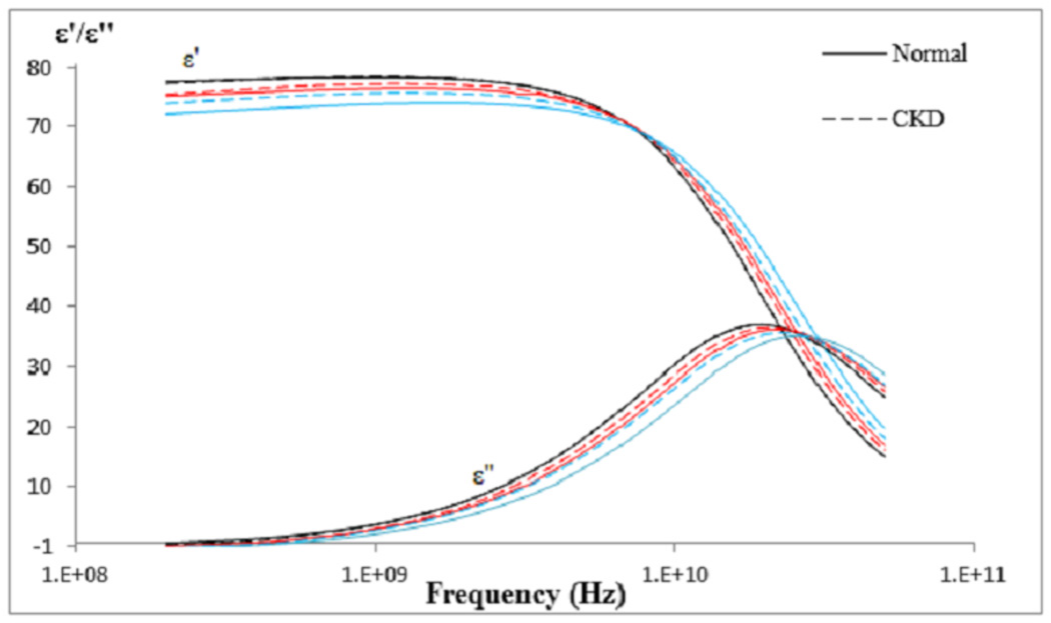
Variation of measured dielectric properties. Mean of measured dielectric properties in terms of dielectric constant and loss factor from normal (solid line) and CKD (dotted line) subjects at respective temperatures of 25°C (black), 30°C (red) and 37°C (blue), and with logarithmic scale of frequency ranging from 0.2 to 50 GHz, were plotted.

Dielectric properties showed different trends with temperature over the frequencies for normal and CKD subjects (Fig 3). A ‘cross-over’ point was observed at about 7 GHz for the dielectric constant and 27 GHz for the loss factor where the dielectric properties were constant over the changes of temperature and subjects. Below this frequency point, the dielectric properties decreased with temperature. Dielectric properties of CKD subjects were found to be higher than those of normal subjects. Meanwhile, dielectric properties increased with temperature above the ‘cross-over’ point. The CKD subjects had lower dielectric properties than those of normal subjects above the frequency point. Generally, the dielectric properties showed more differences between the normal and CKD subjects as the temperature increased from 25°C to 37°C.

The mean and standard deviation values of dielectric properties for normal and CKD subjects at three different temperatures and different microwave frequencies are presented in Table 3. p-value represents the statistical comparison of dielectric properties between normal and CKD subjects at the respective frequency. Stronger statistically significant differences were found between normal and CKD subjects in dielectric constant compared to loss factor. Significant differences in dielectric properties were found at most of the frequencies for 30°C and 37°C, but not for 25°C.

**Table 3:** Mean and standard deviation of dielectric properties for normal and CKD subjects at different microwave frequencies.

*f* (GHz)	25°C				30°C				37°C			
	Normal		CKD		Normal		CKD		Normal		CKD	
	ε’	ε”	ε’	ε”	ε’	ε”	ε’	ε”	ε’	ε”	ε’	ε”
**0.2**	73.75 ± 0.03	0.05 ± 0.44	76.78 ± 0.98	-0.66 ± 2.82	74.66 ± 1.19	-0.44 ± 0.43	75.07 ± 1.83	-1.13 ± 3.0	71.66a ± 1.36	-0.67 ± 0.52	73.35a ± 2.42	-0.99 ± 3.00
**0.4**	77.57 ± 0.64	0.81^a^ ± 0.36	77.53 ± 0.51	0.36^a^ ± 1.06	75.33^a^ ± 0.99	0.16 ± 0.54	76.00^a^ ± 1.25	-0.16 ± 1.26	72.47^a^ ± 1.17	-0.28 ± 0.55	74.24^a^ ± 2.20	-0.28 ± 1.18
**0.6**	77.79 ± 0.48	1.64^a^ ± 0.38	77.85 ± 0.32	1.34^a^ ± 0.77	75.70^a^ ± 0.76	0.91 ± 0.59	76.48^a^ ± 1.05	0.81 ± 0.92	72.93^a^ ± 0.97	0.34 ± 0.60	74.75^a^ ± 2.13	0.51 ± 0.93
**0.8**	77.86 ± 0.39	2.47 ± 0.37	77.94 ± 0.22	2.25± 0.61	75.90^a^ ± 0.59	1.63 ± 0.59	76.67^a^ ± 1.03	1.62 ± 0.93	73.22^a^ ± 0.81	0.95^a^ ± 0.64	74.98^a^ ± 2.13	1.29^a^ ± 0.87
**1**	77.91 ± 0.28	3.27 ± 0.36	78.00 ± 0.24	3.12 ± 0.55	76.01^a^ ± 0.49	2.40 ± 0.57	76.78^a^ ± 1.01	2.51 ±0.68	73.39^a^ ± 0.66	1.59^a^ ± 0.65	75.12^a^ ± 2.14	2.06^a^ ± 0.80
**3**	76.61 ± 0.05	10.91 ± 0.20	76.55 ± 0.43	10.91 ± 0.38	75.05^a^ ± 0.50	9.27^a^ ± 0.31	75.99^a^ ± 1.37	9.81^a^ ± 0.86	73.16^a^ ± 0.18	7.47^a^ ± 0.41	74.47^a^ ± 1.98	8.63^a^ ± 1.57
**5**	73.74 ± 0.04	17.70 ± 0.12	73.76 ± 0.11	17.68 ± 0.14	73.06^a^ ± 0.05	15.33^a^ ± 0.22	73.26^a^ ± 0.48	16.11^a^ ± 1.10	71.63^a^ ± 0.12	12.63^a^ ± 0.28	72.36^a^ ± 1.03	14.35^a^ ± 2.37
**10**	62.81 ± 0.03	29.92 ± 0.06	62.82 ± 0.08	29.92 ± 0.07	64.24^a^ ± 0.06	26.98^a^ ± 0.12	63.71^a^ ± 0.68	28.0^a^ ± 1.44	64.39^a^ ± 1.08	23.24^a^ ± 0.15	64.40^a^ ± 1.07	25.52^a^ ± 3.26
**30**	26.47 ± 0.03	33.30 ± 0.04	26.45 ± 0.18	33.29 ± 0.09	29.87^a^ ± 0.12	34.04 ± 0.11	28.53^a^ ± 1.76	33.89 ± 0.45	34.28^a^ ± 0.11	34.31^a^ ± 0.12	31.60^a^ ± 3.95	34.02^a^ ± 0.37
**50**	14.53 ± 0.09	24.60 ± 0.04	14.48 ± 0.54	24.51 ± 0.38	16.49^a^ ± 0.13	26.31^a^ ± 0.07	15.58^a^ ± 1.25	25.64^a^ ± 1.14	19.32^a^ ± 0.09	28.27^a^ ± 0.09	17.56^a^ ± 2.63	26.88^a^ ± 2.10

The data presented were the mean dielectric properties in terms of dielectric constant (ε’) and loss factor (ε”) for normal (n = 40) and CKD (n = 43) subjects ± standard deviation (S.D.) at 25°C, 30°C, and 37°C, respectively, and at different frequencies with logarithmic scale. In order to test the significant differences in dielectric properties between normal and CKD subjects, data were analyzed using one-way ANOVA test. “p-value” was considered as significant if *p*-value < 0.05.

^a^p <0.05 in comparison between normal and CKD subjects’ dielectric properties at the same frequency.

### Effect of Temperature on Dielectric Properties

Statistically significant differences (p<0.05) in dielectric properties were observed across the temperatures for normal subjects and CKD subjects. In fact, the urine temperature may vary between the body and room temperature in the process of measurement. Temperature variation is important for dielectric properties measurement. Since > 90% of human urine is water content, the relaxation process of water could be applied in the presence of an electromagnetic field. Wentholt et al. [25] reported that dielectric properties of pure water change with temperature, due to the stretching of intramolecular hydrogen bonds between water molecules. Hence, relaxation frequency was observed to increase when the temperature rose from 25°C to 37°C (Fig 3). In the comparison of relaxation frequency between normal urine and pure water [26], normal urine had higher relaxation frequency at 25°C and 30°C (Table 4). This may be due to the combination of the effects of ionic conductance (eg. ionic salts), molecular Brownian movement (eg. urea and creatinine) and hydrogen bonds in urine when the temperature changes that shorten the relaxation time (τ = 1/2пf_c_). The increment in Brownian movement and ion mobility decreased the viscosity of solution when the temperature increased. Thus, relaxation frequency is inversely proportional to the viscosity of the solution [24].

**Table 4:** Comparison of relaxation frequency between urine and pure water.

Temperature (°C)	Relaxation Frequency (GHz)	
	Normal Urine (This study)	Pure water [26]
**25**	19.60	18.56
**30**	22.20	21.65

The comparison relaxation frequency of normal urine with pure water from Ellison [26] at 25°C and 30°C, respectively, are shown.

### Effect of Protein on Dielectric Properties

Statistically significant differences in dielectric properties were reported between normal subjects and CKD subjects (p<0.05). The T-test results showed no statistically significant differences (p>0.05) in creatinine, urea and salt ions (Cl^−^, Na^+^ and K^+^) between normal and CKD subjects. The presence of protein in CKD subjects caused the dielectric properties to change significantly (p<0.05), especially at 30°C and 37°C (Table 5).

**Table 5:** F number and p-value of dielectric properties across proteinuria levels at different microwave frequencies.

Frequency (GHz)	25°C				30°C				37°C			
	ε’		ε”		ε’		ε”		ε’		ε”	
	F Number	p- Value	F Number	p- Value	F Number	p- Value	F Number	p- Value	F Number	p- Value	F Number	p- Value
**0.2**	0.288	0.834	2.533	0.115	1.107	0.107	1.135	0.346	5.997	<0.01^b^	1.108	0.359
**0.4**	1.041	0.385	6.574	0.012b	4.267	<0.01^b^	1.575	0.189	8.242	<0.01^b^	1.672	0.165
**0.6**	1.077	0.370	4.760	0.032	6.600	<0.01^b^	1.527	0.202	9.293	<0.01^b^	2.450	0.053
**0.8**	1.084	0.367	3.657	0.059	7.065	<0.01^b^	1.815	0.134	8.994	<0.01^b^	3.490	0.11
**1**	0.314	0.815	2.039	0.157	7.730	<0.01^b^	1.997	0.103	8.819	<0.01^b^	4.893	<0.01^b^
**3**	1.046	0.383	0.016	0.899	6.425	<0.01^b^	7.993	<0.01^b^	6.421	<0.01^b^	8.844	<0.01^b^
**5**	0.773	0.516	0.376	0.542	3.804	<0.01^b^	8.521	<0.01^b^	8.014	<0.01^b^	8.349	<0.01^b^
**10**	2.587	0.067	0.030	0.862	7.651	<0.01^b^	7.806	<0.01^b^	7.272	<0.01^b^	7.728	<0.01^b^
**30**	0.300	0.585	1.956	0.109	7.740	<0.01^b^	3.779	<0.01^b^	7.242	<0.01^b^	9.598	<0.01^b^
**50**	1.391	0.245	1.436	0.230	6.466	<0.01^b^	6.013	<0.01^b^	6.681	<0.01^b^	17.566	<0.01^b^

In order to test the significant differences in dielectric properties between CKD subjects with different proteinuria levels, data were analyzed using One-way ANOVA test. The p-value was generated from the F number based on the difference and sample variations. “p-Value” was considered as significant if *p*-value < 0.05.

^b^p <0.05 in comparison of dielectric properties between CKD subjects with different proteinuria levels at the same frequency.

**Fig 4:**
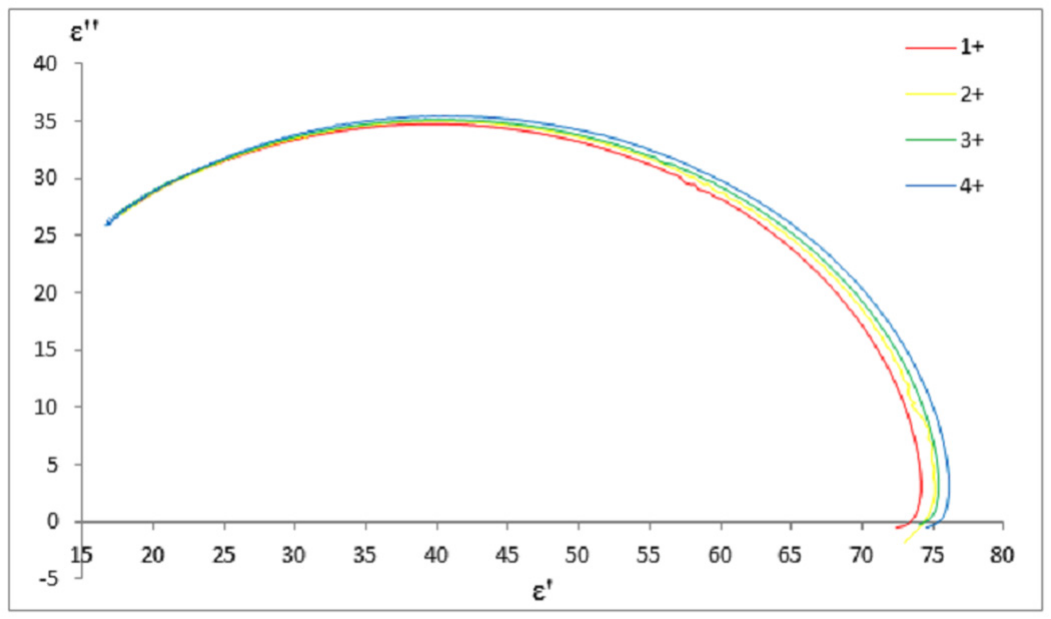
Cole-Cole diagram of different proteinuria levels at 37°C. The graph was plotted to show the changing dielectric properties with different proteinuria levels of CKD subjects at 37°C.

Observable changes in dielectric properties were seen with proteinuria levels at 37°C (Fig 4). Pearson correlation test showed positive correlation between proteinuria and dielectric properties [r_dielectric constant_ (83) = 0.568, p < 0.01; r_loss factor_ (83) = 0.564; p < 0.01] below the “cross-over” frequency point, while negative correlation [r_dielectric constant_ (83) = −0.535, p < 0.01; r_loss factor_ (85) = −0.505; p < 0.01] was found above the “cross-over” frequency point at 37°C. Less correlation was found at 25°C.

Relaxation frequency was shifted to lower frequencies when proteinuria levels increased. As the concentration of protein increases, it decreases the bulk water concentration that causes slower relaxation time scale to replace the faster relaxation time scale of bulk water [15]. Previous studies had reported the changes in dielectric properties of extracted protein solutions in radio [13, 14] and microwave frequencies [27]. In this study, we observed that proteinuria had similar effects with protein solution on changes in dielectric properties. Abdalla et al. [6] pointed out that although protein may not be as massive as other components in complex biological solutions such as blood, additional substances significantly alter the electrical properties of the solution. Desouky [28] discovered that glucose in blood (diabetes mellitus) drastically changed the dielectric properties of blood. Protein-bound water affects the linear conduction rather than the oscillation motion in biological solutions, and this causes drift scatter motion along the electrical field side that changes the overall dielectric properties [6, 12]. Thus, measurement of dielectric properties could be used to differentiate between normal urine and the urine of CKD patients.

### Comparison with Debye Model

**Fig 5:**
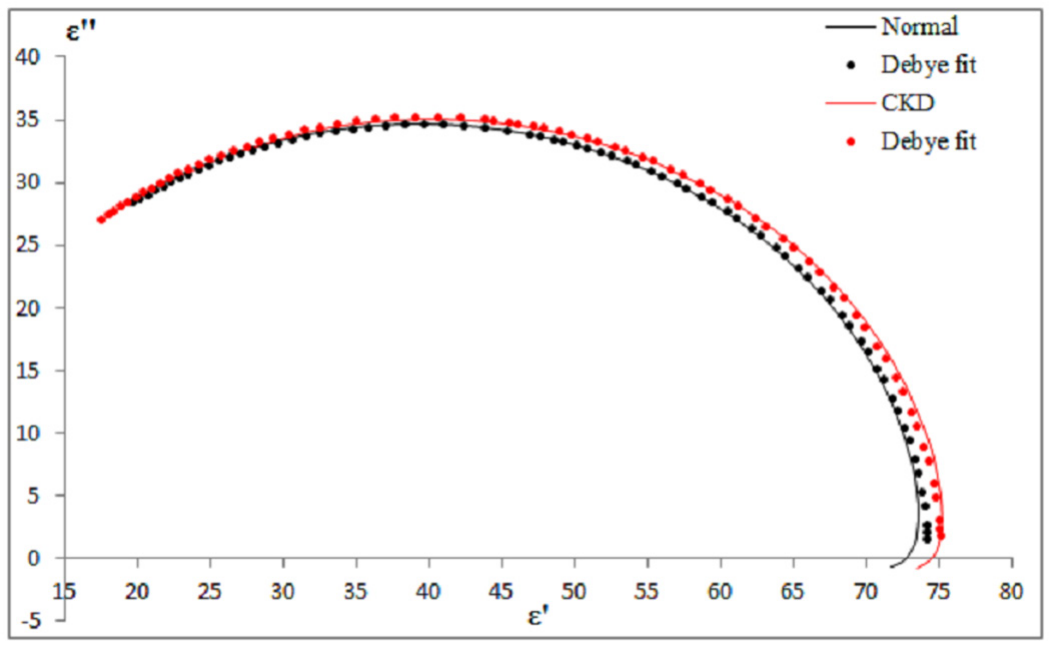
Cole-Cole diagram of experimental and Debye fit data. The graph were plotted to demonstrate variation between experimental and theoretical data for normal and CKD urine at 37°C. The dielectric properties of experimental data were compared to the theoretical data calculated from the Debye model.

Limited comparative data are available on the dielectric behavior of urine. A main dispersion was observed at the measured frequency between 0.2 GHz and 50 GHz. Hence, it is sufficient to model experimental dielectric data with single-pole Debye model using Eq (1). Comparisons of experimental data with Debye data at 37°C are represented using Cole-Cole diagram in Fig 5. Overall, experimental data were found to closely match the single-pole Debye model for normal and CKD subjects. The deviations were mostly observed at low frequencies with about 2% ~3%. This may be due to the instability of the dielectric system to measure low frequencies at below 1 GHz. However, the variations were within the acceptable range of standard error of ± 5%.

According to Fig 5, observable different static permittivity, ε_s_ between normal and CKD subjects were obtained. Infinite frequency permittivity, ε_∞_ was assumed reaching at a constant for normal and CKD although it was not within our measured frequency range. All fitted Debye parameters were calculated using Eq (1), and are displayed in Table 6. ε_∞_ was approximately constant (ε_∞_ ≈ 5) across all the proteinuria levels and temperatures. Theoretically, this indicates water content of urine remained unchanged for all the subject groups. Peyman and Gabriel [9], [29] mentioned that the variation of about 25% on the value of ε_∞_ has very little effect on the other fitted parameters. Δε and τ increased with proteinuria level, while decreased with temperature. The changes were observed to be stronger at a higher temperature (37°C). Dielectric properties of proteinuria were certainly more prominent in body temperature (37°C) rather than in room temperature (25°C). Static conductivity, σ_s_ was negligible and uncertain at low level of proteinuria. Conductivity of urine was relatively small in a constant field as biological solution is quoted with only about 0.9% of salt content [30, 31]. Slight increase of σ_s_ was observed with temperature and proteinuria level reaching 3+ and 4+, respectively. However, this remains a challenge for conductivity determination from measured spectra at limiting low frequencies.

**Table 6:** Debye dielectric parameters of different proteinuria levels at 25°C, 30°C and 37°C.

Proteinuria level	25°C					30°C					37°C				
	ε_∞_	Δε	τ (ps)	σ_s_ (S/m)	RMSPE (%)	ε_∞_	Δε	τ (ps)	σ_s_ (S/m)	RMSPE (%)	ε_∞_	Δε	τ (ps)	σ_s_ (S/m)	RMSPE (%)
0 (Normal)	4.99	72.75	8.20	0.02	0.22	4.98	71.31	7.15	0.02	0.19	4.94	69.30	6.11	0.06	0.28
1+	5.06	72.83	8.23	0.01	0.17	4.86	71.87	7.26	0.01	0.22	4.89	69.83	6.35	0.02	0.18
2+	5.03	73.17	8.26	0.01	0.48	4.96	72.26	7.43	0.02	0.47	4.92	70.56	6.73	0.06	0.45
3+	4.97	73.19	8.27	0.05	0.18	5.02	72.59	7.64	0.07	0.21	5.08	71.55	7.11	0.08	0.26
4+	5.02	73.22	8.30	0.11	0.20	4.93	73.01	7.96	0.12	0.42	5.02	72.91	7.63	0.14	0.39

Debye dielectric parameters of different proteinuria levels were calculated by fitting experimental data into the Debye model. The data presented were the mean of infinite frequency permittivity (ε_∞_), magnitude of dispersion (Δε), relaxation time (τ) and static conductivity (σ_s_) of respective proteinuria levels at respective temperatures of 25°C, 30°C and 37°C. The lowest root mean square percentage error (RMSPE) between the differences of experimental value and the value obtained from the model were determined for fitting.

## Conclusions

We have investigated the dielectric properties of urine in normal subjects and subjects with CKD at microwave frequency between 0.2 GHz and 50 GHz. There were statistically significant differences in the dielectric properties of CKD subjects compared with normal subjects. The dielectric properties of CKD subjects were higher and lower than those of normal subjects for below and above “cross-over” frequency points at 37°C and 30°C, respectively. Pearson correlation test showed significant positive and negative correlation between proteinuria level and dielectric properties below and above the “cross-over” frequency points, respectively. The experimental data closely matched the single-pole Debye model. The relaxation of different proteinuria levels were described using fitted Debye parameters. The relaxation dispersion and relaxation time increased with the proteinuria level, while decreased with the temperature. As for static conductivity, it increased with proteinuria level and temperature.
